# Supplementary material for: Mechanistic insight on water dissociation on pristine low-index TiO2 surfaces from machine learning molecular dynamics simulations
Source: Nat Commun. 2023 Oct 2;14:6131. doi: 10.1038/s41467-023-41865-8 (PMC10545769; doi:10.1038/s41467-023-41865-8)
Supplement: Supplementary file 1 — Supplementary Information [file 41467_2023_41865_MOESM1_ESM.pdf]

Supplementary Information:
Mechanistic insight on water dissociation on pristine low-index TiO_2 surfaces from machine learning molecular dynamics simulations

Zezhu Zeng,[1] Felix Wodaczek,[1] Keyang Liu,[2] Frederick Stein,[3,4] Jürg Hutter,[3] Ji Chen,[2,5,6] and Bingqing Cheng[1,*]

[1]*The Institute of Science and Technology Austria, Am Campus 1, 3400 Klosterneuburg, Austria*

[2]*School of Physics, Peking University, Beijing 100871, People's Republic of China*

[3]*Department of Chemistry, University of Zurich, Winterthurerstrasse 190, 8057 Zurich, Switzerland*

[4]*Center for Advanced Systems Understanding (CASUS), Helmholtz-Zentrum Dresden, Rossendorf (HZDR), Untermarkt 20, 02826 Görlitz, Germany*

[5]*Interdisciplinary Institute of Light-Element Quantum Materials and Research Center for Light-Element Advanced Materials, Peking University, Beijing, China.*

[6]*Frontiers Science Center for Nano-Optoelectronics, Peking University, Beijing, China*

(Dated: September 15, 2023)

* bingqing.cheng@ist.ac.at

Simulation details of optB88-vdW AIMD

We used the CP2K package [1] with a planewave cutoff of 350 Rydberg (Ry) and optB88-vdW functional to perform the NVT MD simulations at 300 K with a timestep of 1 fs. The total run time is 15 ps, and we collected data after 5 ps. For this system, a simulation time of 15 ps is not ergodic, but the aim here is not to sample the equilibrium distribution, but to benchmark the MLP under the same setting. The system contains 64 water molecules, and about 200 TiO_2 atoms. We consider pristine anatase (101), (001), and (110) surfaces and rutile (110), (101), (001), and (100) surfaces, and all these surfaces with defects (by removing 1 or 4 TiO_2 formula units on the surface, see Fig. S1). The initial configuration is a snapshot that has been relaxed using the MLP.

Convergence tests of the SCAN functional

A high planewave energy cutoff for the SCAN functional was used in recent studies on similar systems [2–4]. Here we thus performed a convergence test based on a small set of TiO_2-water configurations using a range of different planewave cutoffs. In Fig. S2 we compare the energies and the forces computed using a 1200 Ry cutoff (x-axes) and 350-1000 Ry cutoff values (y-axes). Although the energies are already well-converged at 350 Ry cutoff, the forces on atoms are slower to converge with respect to the cutoff. Our benchmark is consistent with the remark in Ref. [3], noting that 1200 Ry was used to ensure that the forces are well-converged.

In the main text, we thus constructed SCAN MLP computed using 1200 Ry cutoff. However, we note that a lower cutoff would be sufficient as well: in an early version of the manuscript we reported the results obtain using SCAN MLPs based on 350 Ry cutoff, and the results are essentially identical. This is because the MLPs can overcome the noise in the training set by acting as an interpolator between different atomic configurations.

Benchmark of the MLP

The predicted lattice constants based on our optB88-vdW MLP with the committee model are in good agreement with both previous calculations and experiments as shown in Table S1. Using the optimized lattice constants, we calculated the lattice energies of the anatase and rutile phases based on the committee optB88-vdW MLP model with four fits. Our MLPs suggest that anatase is found to be more stable than rutile by 0.025 ± 0.005 eV/atom. Here the errors are based on the standard

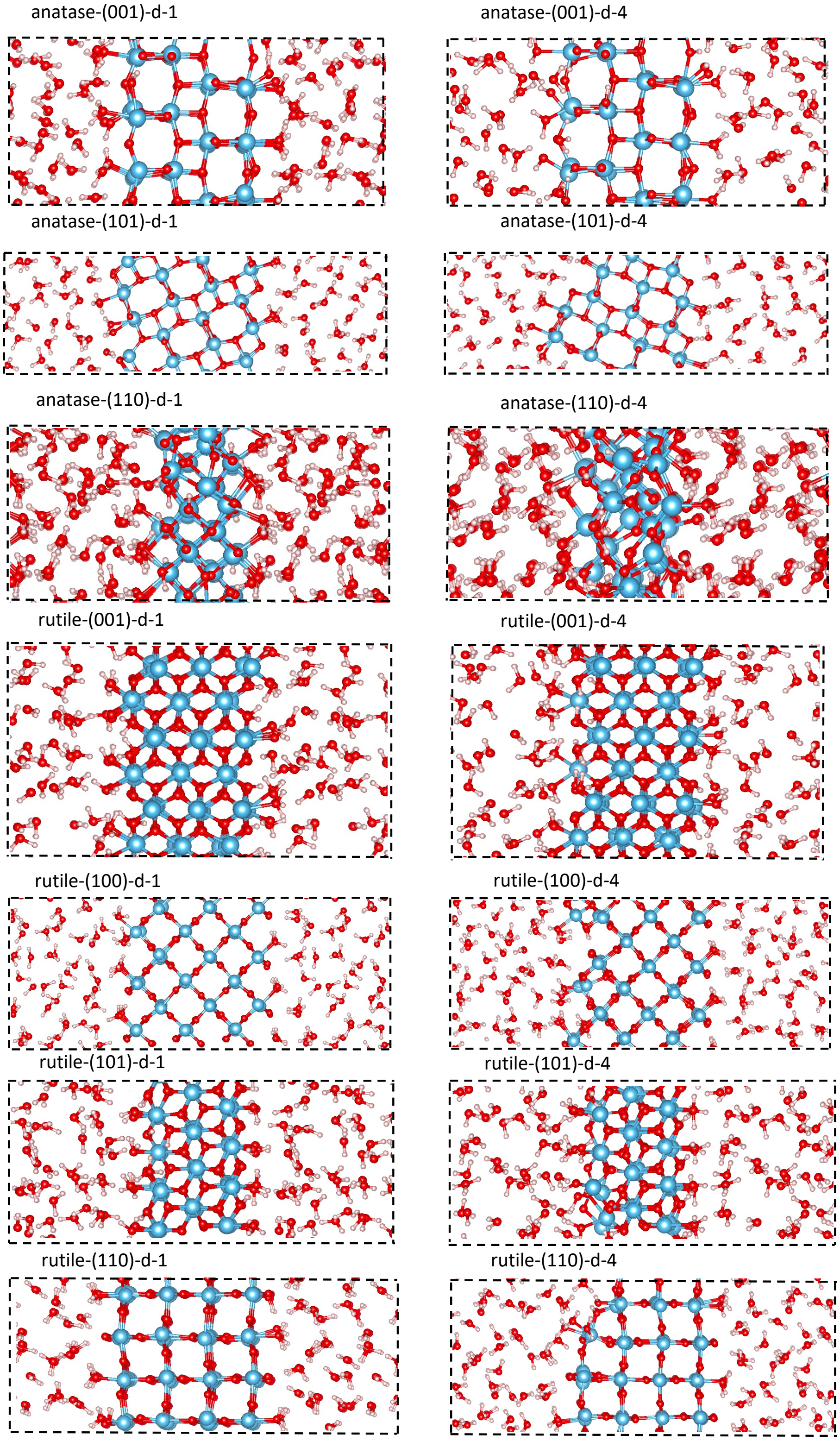

FIG. S1: Illustration of the seven water-interface systems with defects removed from the left surface with 1 (left panel) or 4 (right panel) TiO_2 formula units.

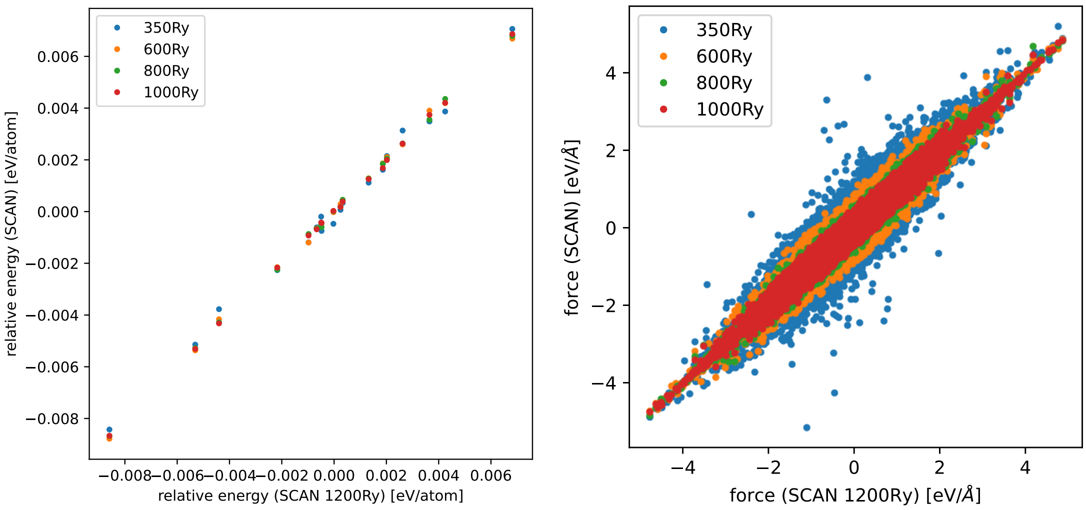

FIG. S2: Parity plots of the energies and the forces of selected TiO_2-water configurations computed using SCAN functional employing a 1200 Ry cutoff (x-axes) and 350-1000 Ry cutoff values (y-axes).

deviations of the estimates from the 4 individual MLPs. This result contradicts experiments, but it is well-known that DFT calculations and experiments give contradicting results regarding the stability of the two phases. This is also consistent with extensive previous DFT calculations that predicted anatase to be more stable than rutile at 0 K by 0.032 eV/atom at the PBE level [5], 0.01 eV/atom at the LDA level [6], and 0.021 eV/atom at the PBEsol level [7]. Our previous study [8] also found anatase to have lower lattice energy then rutile at 0 K and ambient pressure, using DFT calculations based on LDA (0.014 eV/atom), PBE (0.048 eV/atom) and PBEsol (0.021 eV/atom). We also performed DFT calculations in this study, and predicted that anatase is slightly more stable than rutile at 0 K by 0.67 meV/atom at the optB88-vdW level.

We calculated surface energies at 0 K for eight surfaces using optB88-vdW DFT (with an energy cutoff of 350 Rydberg) and the optB88-vdW MLPs with the committee model (see Table S2). The MLPs can reproduce the DFT surface energies very well. The MLP results also agree well with previous DFT calculations (see Table S2 for the comparison). The surface energies are computed using the difference in energies between bulk lattice and systems with relaxed surfaces.

We further compared (see Fig. S3) between the MLP and DFT energies and atomic forces using configurations generated by the MLP-based metadynamics simulations for all seven (anatase (100), (101) and (110), rutile (001), (011), (100) and (110)) TiO_2-water interface systems. For the energy comparison, we see that the raw data extracted from the DFT calculations exhibit a very

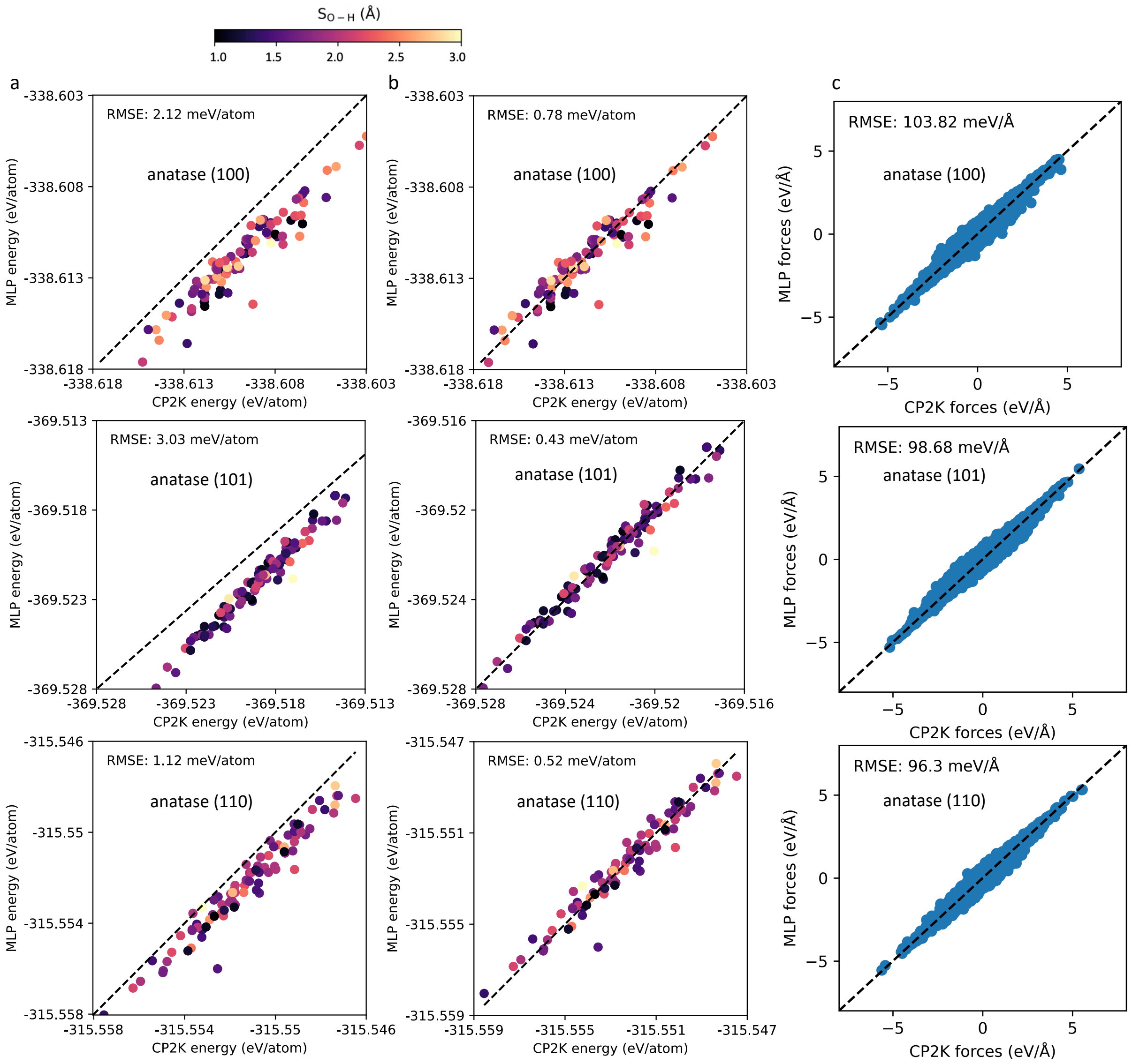

FIG. S3: (a) Raw and (b) adjusted energy comparisons of randomly selected 100 configurations for seven TiO_2 surfaces calculated separately from DFT (optB88-vdW functional with energy cutoff of 350 Rydberg) and optB88-vdW MLP. Each point is color-coded based on the collective variable (S_{O-H}) of the configuration it represents. (c) Atomic forces comparison of the same configurations for seven TiO_2 surfaces calculated separately using DFT and MLP.

small energy offset compared to that from MLP calculations. This offset could be attributed to the smaller system size and TiO_2/water ratio used in the training set. Note that the energy offset for each interface system does not affect the free energy surface or the dynamics of the water adsorption and dissociation process. We also show comparison after adjusting for this offset, and observe excellent agreements between MLP and DFT energies. The maximum root mean square error (RMSE) for the energy after adjusting the energy offset is from the anatase (100) surface, which

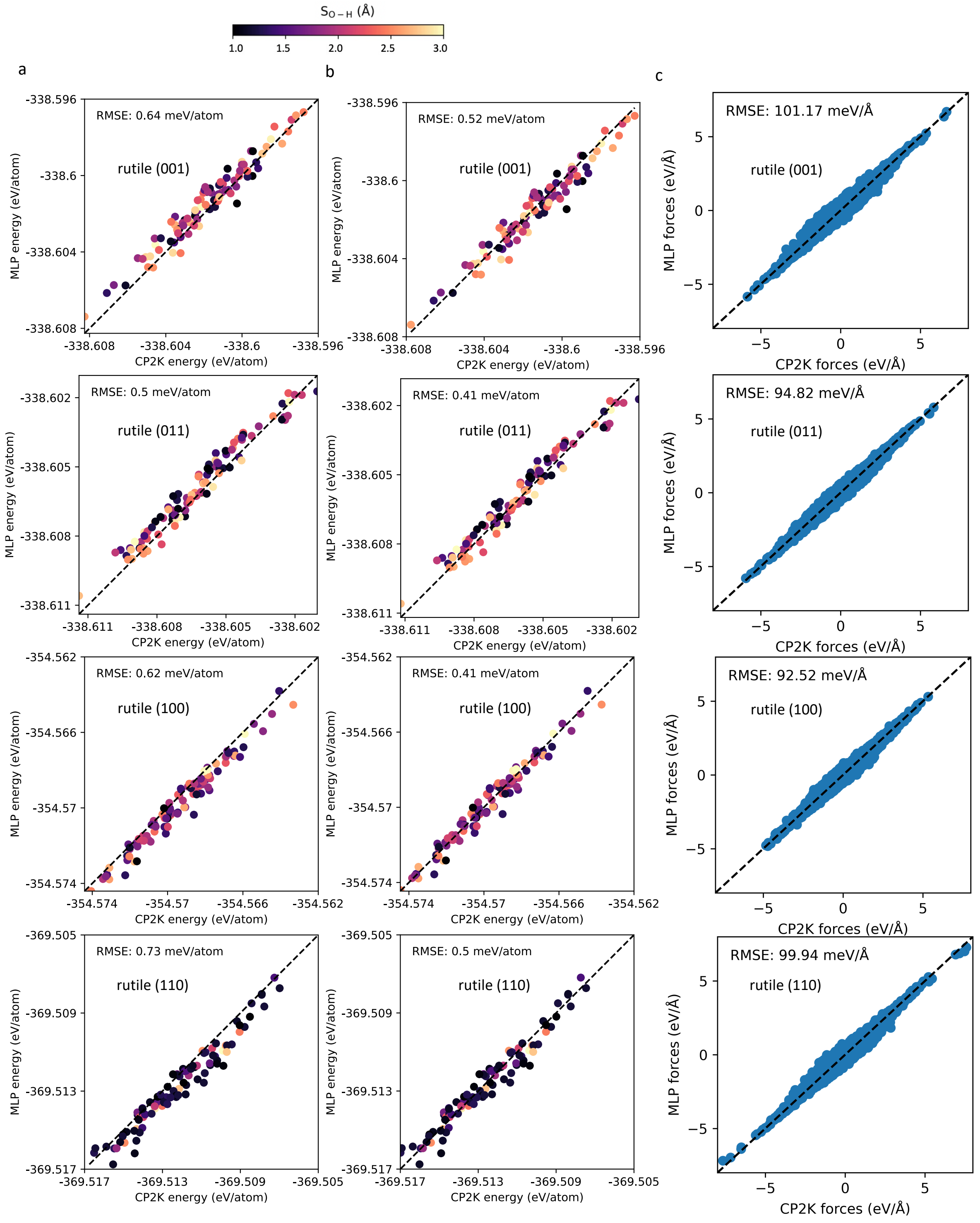

FIG. S3: (a) Raw and (b) adjusted energy comparisons of randomly selected 100 configurations for seven TiO_2 surfaces calculated separately from DFT (optB88-vdW functional with energy cutoff of 350 Rydberg) and optB88-vdW MLP. Each point is color-coded based on the collective variable of the configuration it represents. (c) Atomic forces comparison of the same configurations for seven TiO_2 surfaces calculated separately using DFT and MLP (cont.).

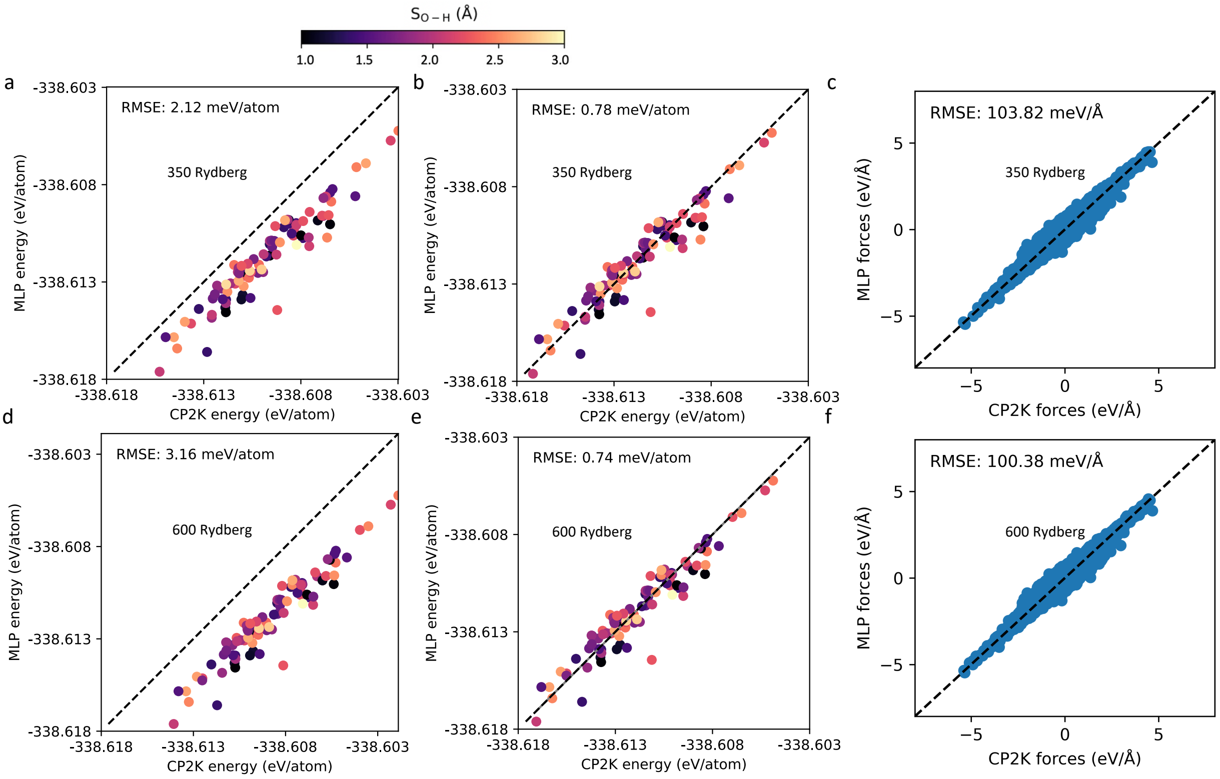

FIG. S4: The comparison of energies and atomic forces of selected 100 configurations of anatase (100) interface system calculated separately from DFT (optB88-vdW functional) and optB88-vdW MLP. (a-c) An energy cutoff of 350 Rydberg was used for single-point DFT calculations. (d-f) An energy cutoff of 600 Rydberg was used for single-point DFT calculations.

is 0.78 meV/atom. Notably, this value is still smaller than the training RMSE of our MLP. For the atomic forces, the maximum RMSE among these seven surfaces is 103.82 meV/Å (again for the anatase (100) surface), which is also lower than the training force RMSE. We also performed DFT calculations on configurations from the anatase (100) surface using the optB88-vdW functional, with an increased energy cutoff of 600 Rydberg. Comparing the results shown in Fig. S4, we observe that the RMSEs of the energy and atomic forces remained largely unchanged, indicating that an energy cutoff of 350 Rydberg is sufficient. These extensive comparisons provide strong evidence of the reliability and accuracy of our MLP in precisely assessing the free energy surface and water dissociation process.

We also benchmarked the optB88-vdW MLPs by comparing it to the optB88-vdW AIMD simulations. Note that the AIMD simulations last for 15 ps, and the sampled distribution is not equilibrated due to the high water dissociation free energy barrier and the slow water diffusion near the surface. We note that the AIMD results are not thermodynamically converged due to the

TABLE S1: Comparison for lattice constants (a, c) of anatase and rutile TiO_2 between experiments and calculations. The errors of the MLP values are from the standard deviations of the estimates from the 4 individual fits.

Materials	functional	a (Å)	c (Å)
anatase-MLP (our work)	optB88-vdW	3.772 ± 0.006	9.761 ± 0.043
anatase-DFT [9]	GGA-PBE	3.787	9.880
anatase-DFT [10]	GGA-PBE	3.820	9.857
anatase-DFT [11]	SCAN	3.77	9.52
anatase-MLP [12]	SCAN	3.80	9.52
anatase-Exp. [13]	-	3.785	9.530
anatase-Exp. [14]	-	3.795	9.514
anatase-Exp. [15]	-	3.782	9.502
rutile-MLP (our work)	optB88-vdW	4.624 ± 0.005	2.987 ± 0.005
rutile-DFT [16]	GGA	4.640	2.980
rutile-DFT [16]	LDA	4.570	2.940
rutile-DFT [17]	LDA	4.554	2.922
rutile-Exp. [18]	-	4.593	2.959
rutile-Exp. [19]	-	4.592	2.958
rutile-Exp. [20]	-	4.587	2.954

TABLE S2: Calculated surface energies (SE, in J/m^2) of eight TiO_2 surfaces based on the DFT (optB88-vdW functional with an energy cutoff of 350 Rydberg) and committee optB88-vdW MLPs. The errors of the MLP values are from the standard deviations of the estimates from the 4 individual MLP fits. Results from literature are presented for comparison.

Surfaces	optB88-vdW-DFT	Our work-MLP	LDA-DFT [6]	PBE-DFT [6]	LDA-DFT [21]
anatase (001)	1.306	1.327 ± 0.006	1.38	0.98	-
anatase (100)	0.876	0.881 ± 0.006	0.96	0.58	0.85
anatase (101)	0.763	0.680 ± 0.005	0.84	0.49	0.97
anatase (110)	1.374	1.421 ± 0.006	-	-	-
rutile (001)	1.353	1.325 ± 0.002	-	-	1.87
rutile (011)	1.236	1.174 ± 0.003	-	-	-
rutile (100)	0.933	0.912 ± 0.006	-	-	1.19
rutile (110)	0.819	0.811 ± 0.004	0.84	0.35	0.91

significant computational time required for DFT calculations spanning several nanoseconds, so we were unable to directly compare the converged density profiles obtained from DFT and MLP calculations. For the sake of benchmarking the MLPs, the simulation setup of the MLP MD was selected to closely resemble the AIMD runs: same initial configurations, same thermodynamic conditions, same time step size and simulation length. The comparison of the density profiles for the oxygen and the hydrogen atoms are in Fig. S5 and Fig. S6. The comparison of the oxygen-oxygen

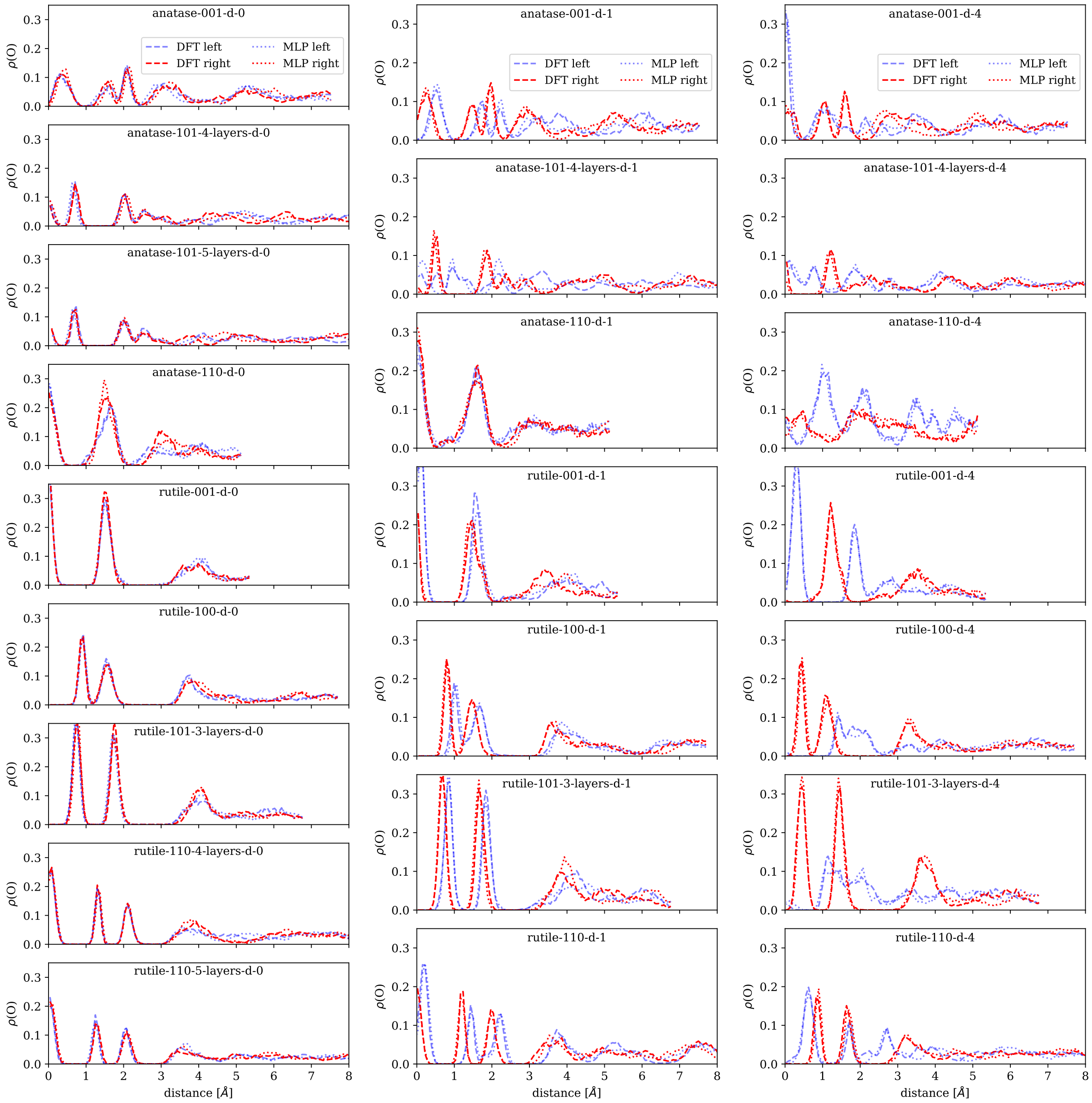

FIG. S5: The density profile $\rho(\mathrm{O})$ of all oxygen atoms. The origin is chosen as the equilibrium position of the Ti atoms closest to the interface. The left and right represent the $\rho(\mathrm{O})$ on the two sides of the TiO_2 slab. The defected surface is always on the left-hand side.

radial distribution functions is shown in Fig. S7.

The orientation of adsorbed water molecules provides another benchmark against which the MLP's accuracy of describing TiO_2-water interface can be measured. The adsorbed water (H_2O-Ti) consists of all water molecules adsorbed on surface Ti atoms, for which the Ti-O distance is within 2.65 Å. Neither the water as a hydrogen bond donor to bridging oxygen nor the OH formed by bridging oxygen was taken into account in our analysis. Each trajectory was divided into three parts

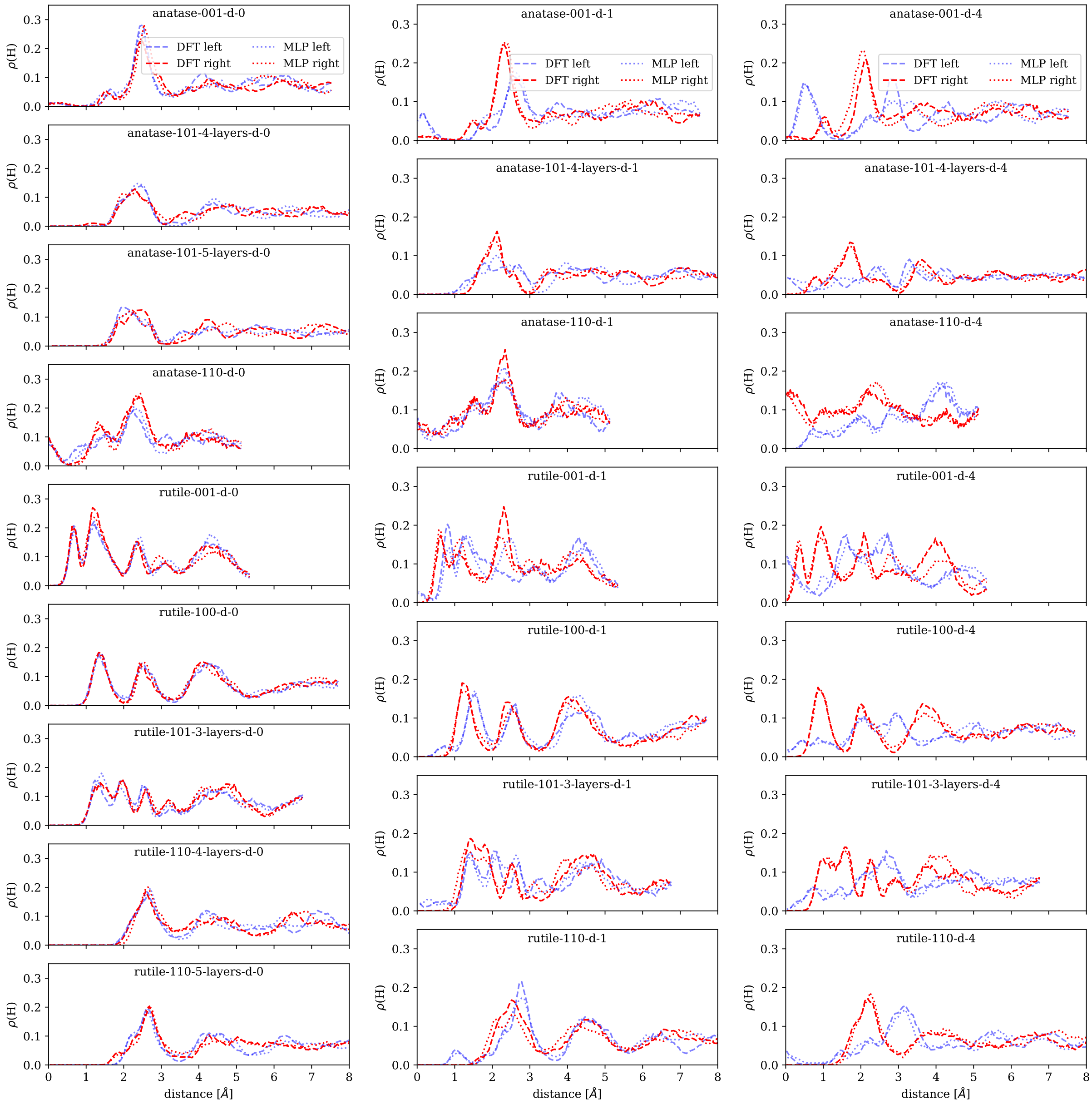

FIG. S6: The density profile $\rho(\mathrm{H})$ of all oxygen atoms. The origin is chosen as the equilibrium position of the Ti atoms closest to the interface. The left and right represent the $\rho(\mathrm{H})$ on the two sides of the TiO_2 slab. The defected surface is always on the left-hand side.

to obtain the average normalized distribution and estimated errors. The orientation distribution varies on different surfaces and can be used as a characteristic description of the TiO_2 surface, which reflects the structure of the surface and interfacial water. Our results (see Fig. S8) shows that well-trained MLP can reproduce very well the DFT MD simulations for various surfaces.

Previous calculations from Schran et al. [22] reported water density profiles on rutile (110) surface based on both AIMD and committee neural network potential (C-NNP) MD simulations

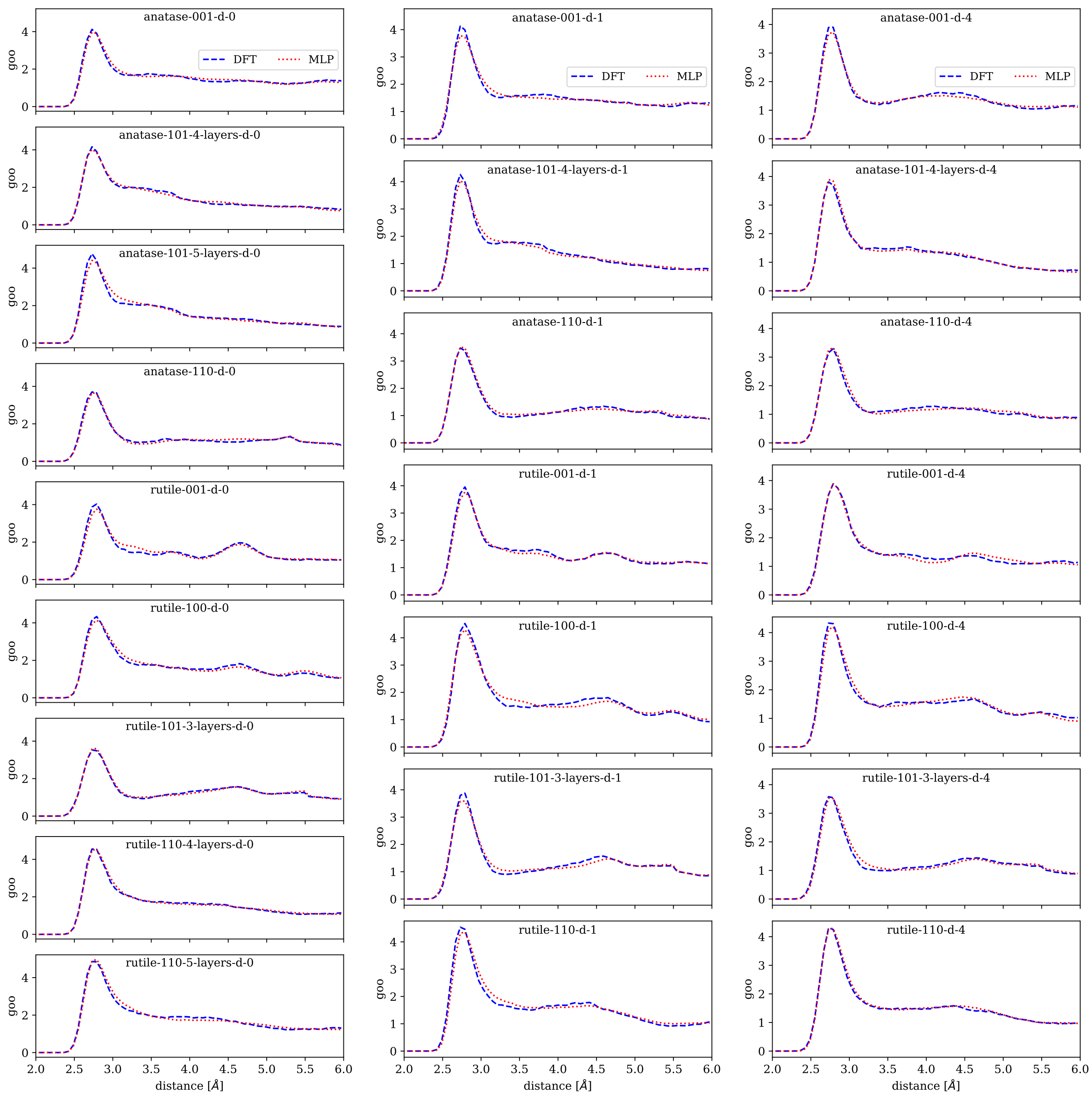

FIG. S7: O-O radial distribution functions (g_{OO}) of all the water molecules in the TiO_2/water interface systems.

with optB88-vdW DFT functional. Andrade et al. [12] also calculated water density profiles on anatase (101) surface on top of both AIMD and deep potential molecular dynamics (DPMD) [23] simulations with SCAN functional. We compared the density profiles and showed the results in Fig. S9 for both the two surfaces, and obtained good agreements. We used the same simulation setup (same temperature, system size) as their simulations.

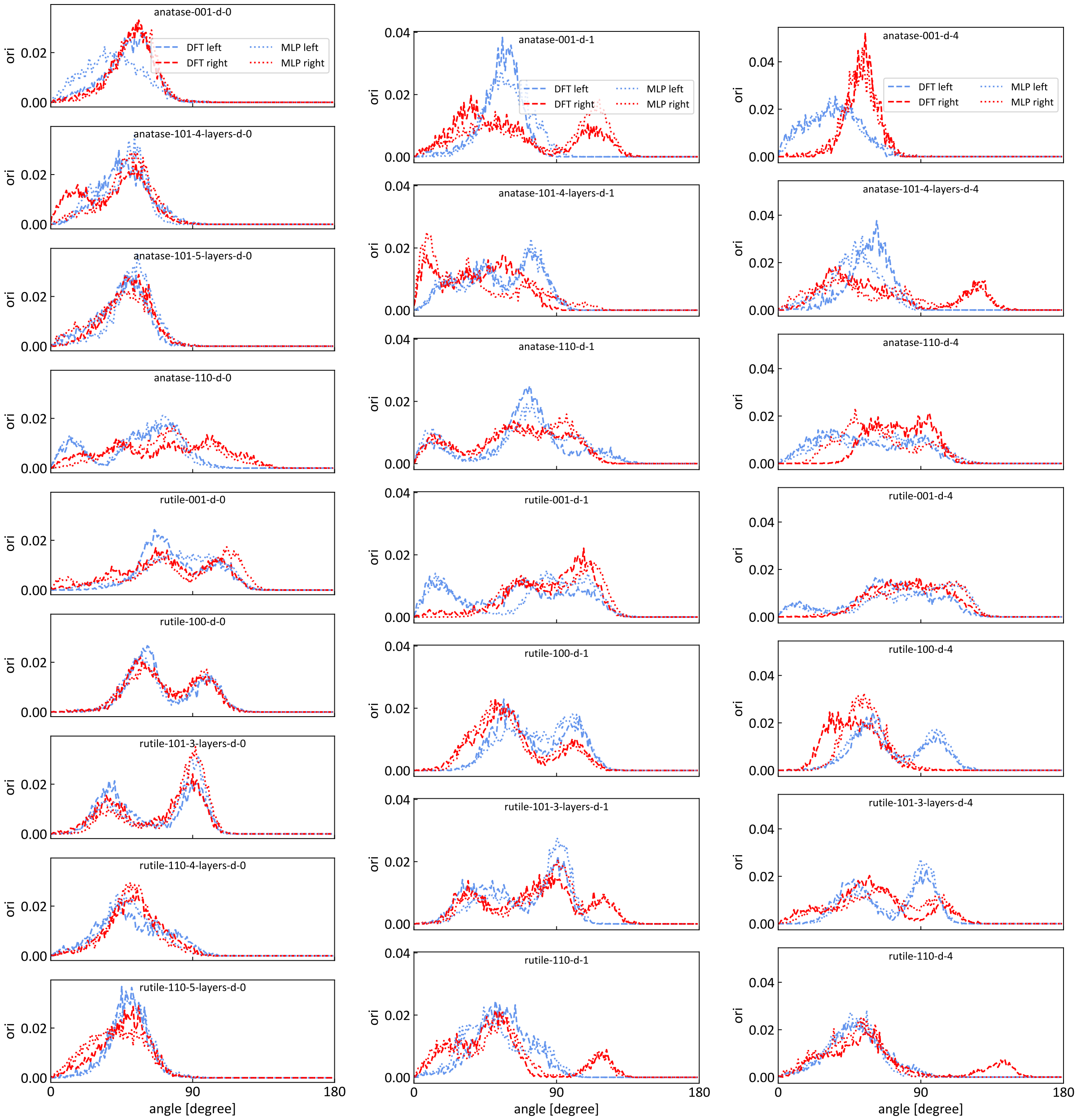

FIG. S8: Normalized orientation distribution (ori) of adsorbed water (H_2O-Ti) calculated using respectively AIMD and MLP MD simulations. Subheadings highlight the polymorph of TiO_2, the exposed lattice surface, the layer thickness, the number of stoichiometric defects and the inclusion of vacuum. The shadow area in each subfigure corresponds to the statistical error of three divided trajectories.

Convergence test on the TiO_2 slab thickness

We performed convergence tests to probe the dependence of water dissociation on the thickness of the TiO_2 slab in simulations. We used optB88-vdW MLP metadynamics simulations to compute the free energy surfaces (FES) of water dissociation (see Fig. S10), on the seven surfaces with

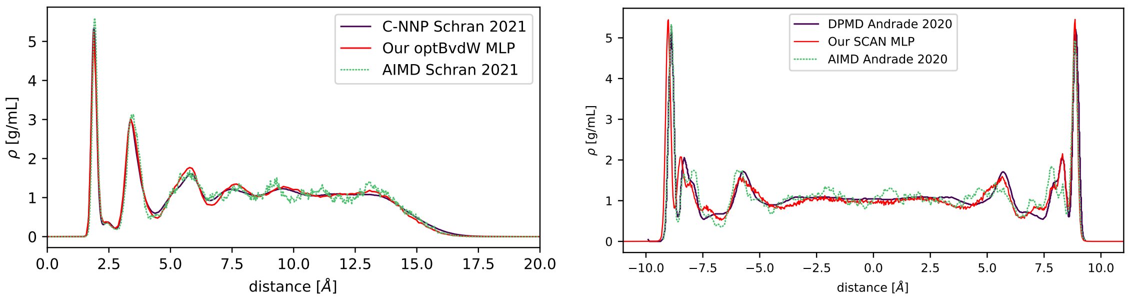

FIG. S9: Left panel: water density profile ρ of rutile (110), compared to previous optB88-vdW AIMD and C-NNP MD results from Schran et al. [22]. Right panel: water density profile ρ of anatase (101), compared to previous SCAN AIMD (about 40 ps long) and DPMD results from Andrade et al. [12].

different slab thickness. For rutile (110), we observed odd-even oscillation for the free energy difference between water molecular adsorption and dissociation, and the oscillation finally subsides after a slab thickness of around 10 layers. The same oscillation was previously oberserved in DFT simulations [24] and MLP MD simulations [4, 25] of rutile (110). The fact that we can reproduce the oscillation behavior at different rutile (110) slab thickness evidences the reliability and generality of our MLPs. As also shown in Fig. S10, for the other six surfaces, no evident odd-even oscillation behavior is observed.

Additional analysis for the MLP simulations

In Table S3 we report the free energy differences (ΔG) between water adsorption and dissociation, and free energy activation barriers ($G^{\star}$) for molecular water to dissociate for the seven water-TiO_2 interfaces. These correspond to the FES values reported in Fig.1 of the main text.

kPCA plots of H environments

In Fig.2 of the main text, we show a subset of the kPCA plots of the hydrogen atomic environments in water-rutile (110) system, and here in Fig. S11 we show all the plots for anatase and rutile facets. The features that we used to color the plots include the classification of H states, the H to its closest Ti distance (H-Ti), H to its closest neighboring H (H-H) and the second closest H distance (H-H^2), H to its closest O in TiO_2 (H-O_t) and its closest O in water (H-O_w), the surface normal of the displacement between H and its closest O (OH_z), the proton transfer coordinates determined by

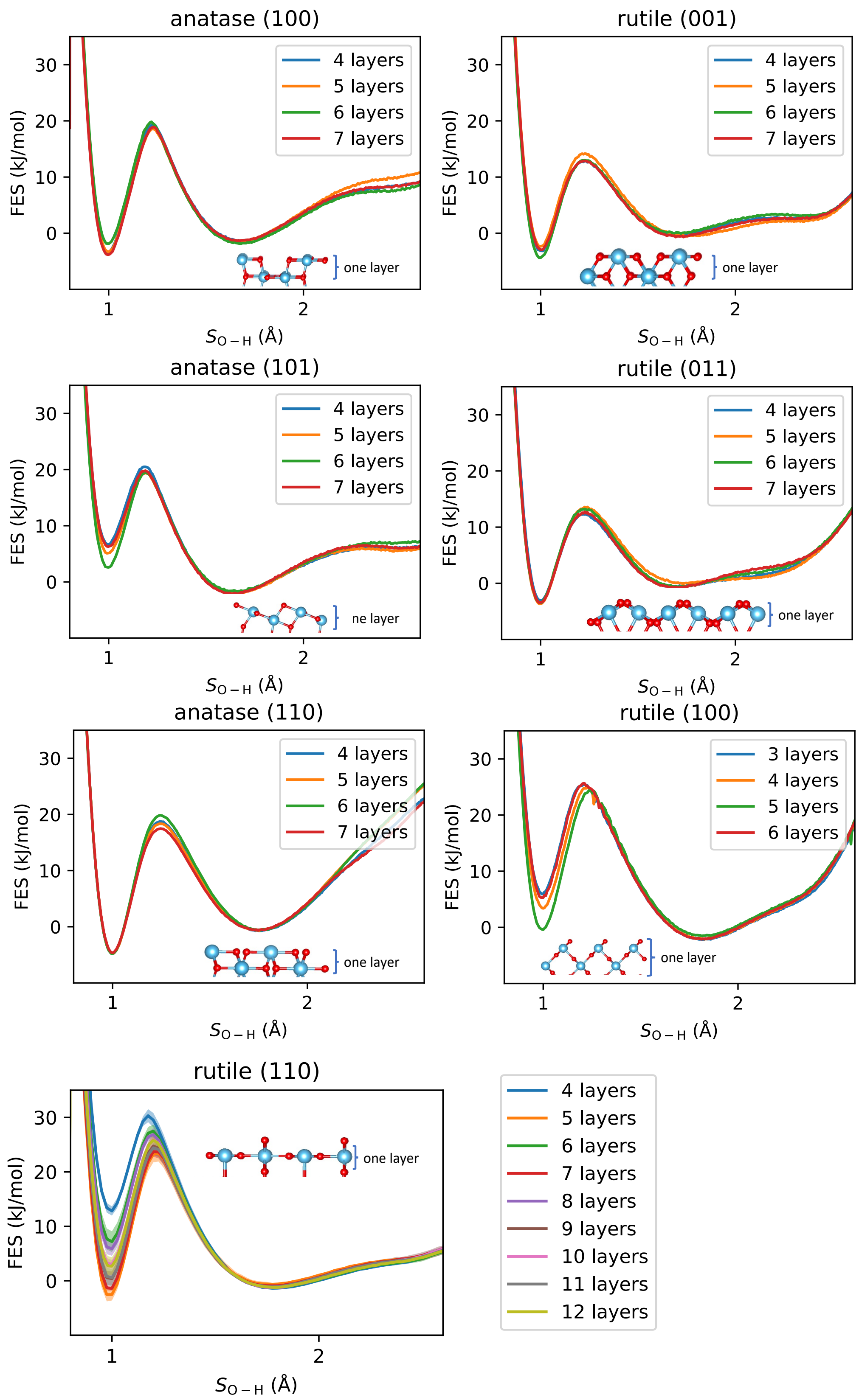

FIG. S10: Free energy surfaces (FES) with different slab thicknesses calculated from optB88-vdW MLP metadynamics simulations for seven surfaces. $S_{\mathrm{O-H}}$ is the minimal distance of an undercoordinated twofold surface O_{2c} atom to any hydrogen in the system. For rutile (110), we used the committee model with four individual fits to estimate the standard deviations (shaded areas).

TABLE S3: Free energy differences (ΔG) between water adsorption and dissociation, and free energy activation barriers ($G^{\star}$) for molecular water to dissociate for the seven water-TiO_2 interfaces. The data is taken from the Fig. 1d of the main text. The errors of the MLP values are from the standard deviations of the estimates from the 4 individual fits.

Surfaces	Functionals					
	SCAN		PBE		optB88-vdw	
	ΔG	$G^{\star}$	ΔG	$G^{\star}$	ΔG	$G^{\star}$
anatase (100)	-1.4 ± 0.9	21.0 ± 1.5	-3.6 ± 0.3	16.2 ± 1.1	-2.9 ± 0.4	19.4 ± 0.8
anatase (101)	8.2 ± 1.7	23.0 ± 2.1	8.0 ± 0.9	20.3 ± 1.5	7.4 ± 1.3	22.7 ± 1.7
anatase (110)	-1.5 ± 3.1	20.2 ± 4.5	-5.3 ± 2.0	15.7 ± 2.1	-2.5 ± 1.2	19.9 ± 1.5
rutile (001)	-5.2 ± 1.6	12.5 ± 2.4	-4.6 ± 0.7	11.5 ± 1.1	-3.7 ± 0.3	13.4 ± 0.4
rutile (011)	-3.0 ± 0.4	14.0 ± 0.6	-2.2 ± 1.1	12.4 ± 2.4	-2.5 ± 0.6	13.4 ± 1.1
rutile (100)	14.0 ± 0.7	31.0 ± 2.3	12.9 ± 0.3	26.3 ± 0.9	14.8 ± 1.3	29.2 ± 3.3
rutile (110)	2.6 ± 1.2	27.5 ± 1.9	5.1 ± 0.6	24.2 ± 2.1	5.2 ± 0.6	26.8 ± 1.5

the positions of the hydrogen, a donor oxygen atom O and an acceptor O′ ($v = d(\mathrm{OH}) - d(\mathrm{O'H})$, and $r_{\mathrm{OO}} = d(\mathrm{OO'})$).

From the kPCA maps in Fig. S11, the whole set of H environments forms well-separated clusters (rather than having a continuous spectrum of environments), suggesting that it is physically meaningful to assign protons to different sites. As the clusters are distinct, we found that a few assignment schemes all work quite well, such as automatic clustering methods (e.g. DBSCAN) and decision-tree methods. Eventually we used a simple decision tree based on the following criteria: if the hydrogen distance to its nearest oxygen in TiO_2 is more than 5 Å, it is classified to be far from the surface ($H_2O^{(>1)}$); instead if the hydrogen distance to its nearest oxygen in TiO_2 is less than 1.25 Å, and the hydrogen distance to its closest neighboring hydrogen (H-H) is more than 1.6 Å, the hydrogen is classified as H adsorbed on the surface O (H-O_t); else if the H-O_t distance is more than 1.85 Å, and the nearest O_w-Ti distance is less than 3 Å, this is a OH adsorbed on Ti (HO-Ti); else if the nearest O_w-Ti distance is less than 3 Å, it is adsorbed H_2O (H_2O-Ti); else the hydrogen is classified as first-layer H_2O ($H_2O^{(1)}$). The same criteria were used for all surfaces. The classification is not very sensitive to the cutoffs used above, as long as they are within a reasonable range.

Additional analysis of proton transfer mechanisms and rates

We use anatase (101) as an example to explain the proton transition matrix as shown in Fig. 3d of the main text. The matrix has a dimension of 5 × 5 as we have classified all hydrogen

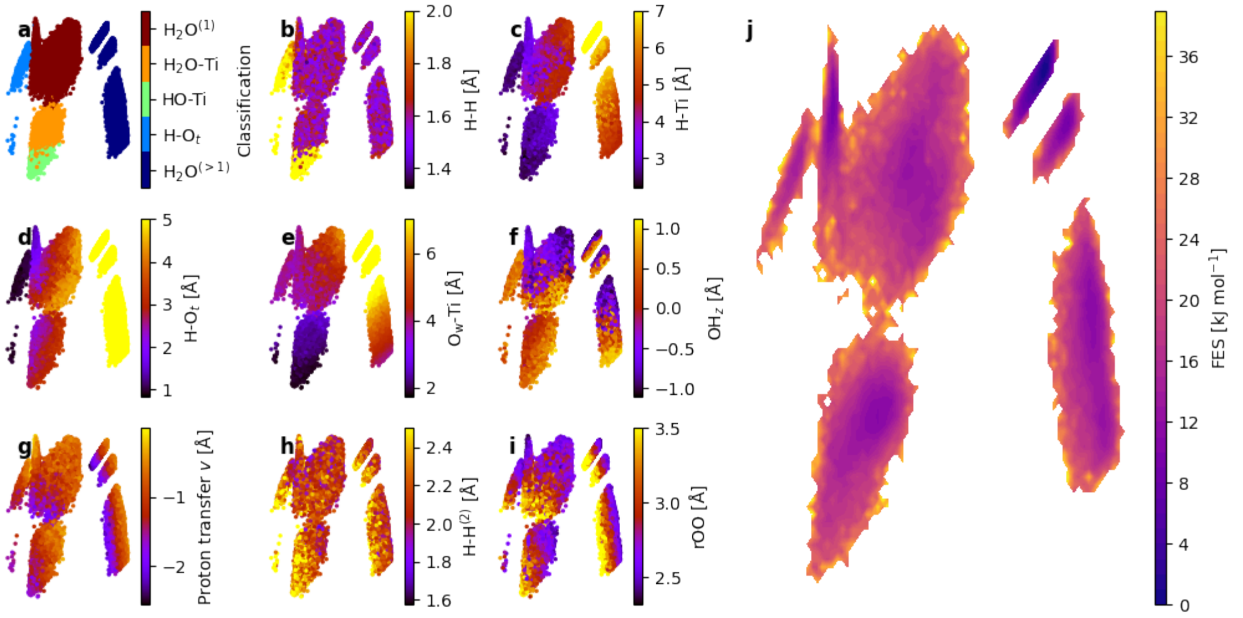

(a) anatase (100)

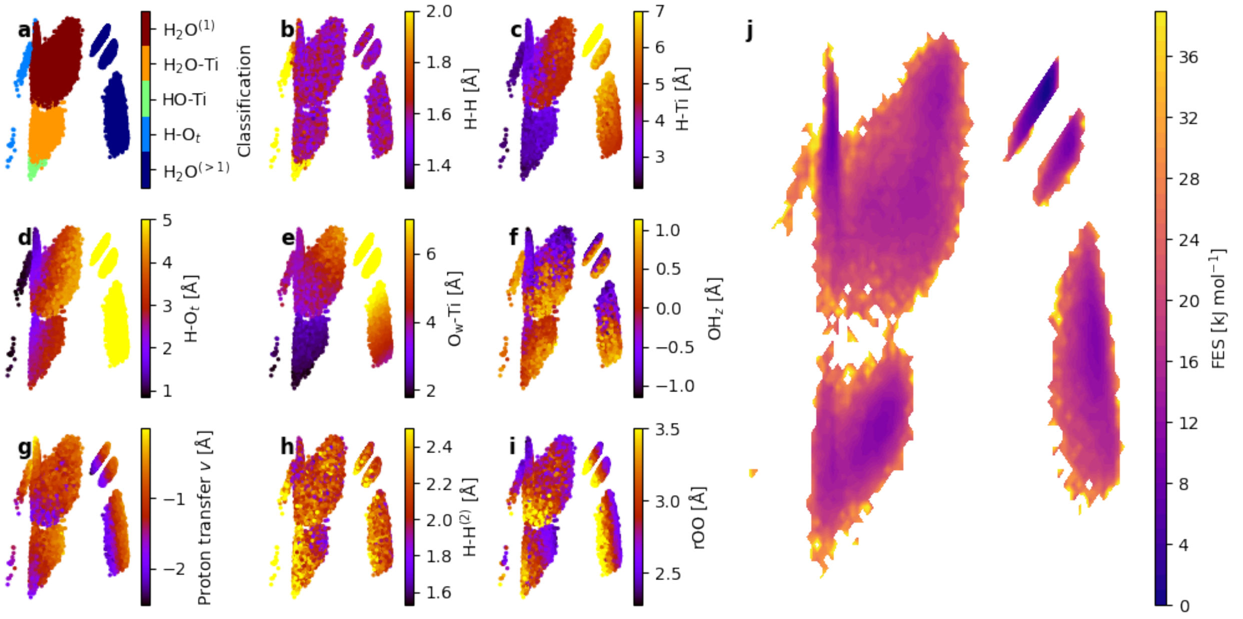

(b) anatase (101)

FIG. S11: (a-i) kernel Principal Component Analysis (kPCA) of the hydrogen atomic environments in various water-anatase TiO_2 systems. (j) The free energy surface (FES) as a function of the two principal axes of the kPCA map of the hydrogen environments.

environments into five states. Since transitions of protons between certain states are not possible, the corresponding matrix elements remain unfilled with color (the probability is equal to zero). For instance, protons from $H_2O^{(>1)}$ to surface O_{2c} (H-O_t). For the one-step mechanism, H_2O-Ti and H-O_t are the two involved states, and the corresponding element is marked as a red star in

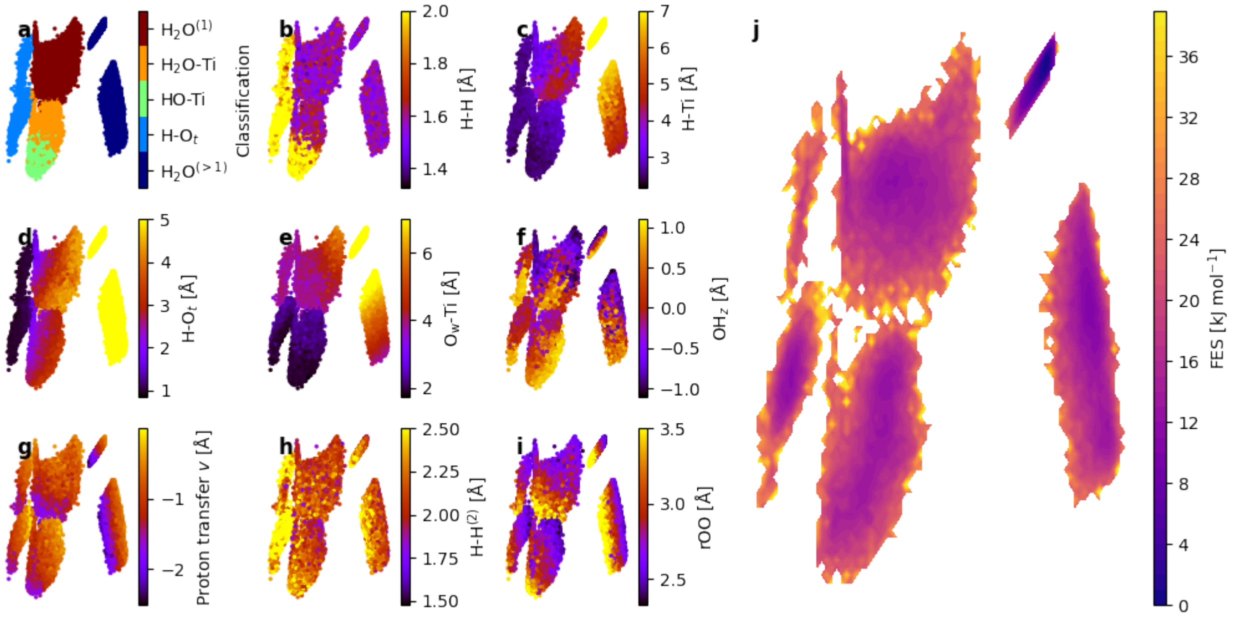

(c) anatase (110)

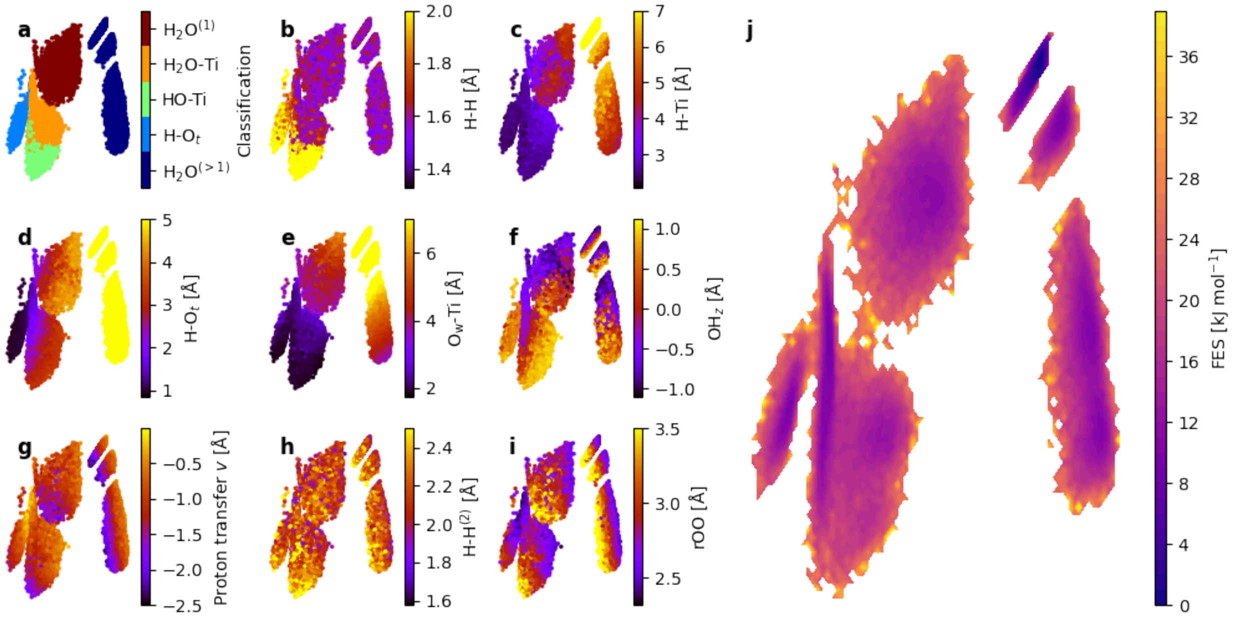

(d) rutile (001)

FIG. S11: (a-i) kernel Principal Component Analysis (kPCA) of the hydrogen atomic environments in various water-anatase/rutile TiO_2 systems. (j) The free energy surface (FES) as a function of the two principal axes of the kPCA map of the hydrogen environments (cont.).

the lower triangle of the matrix. From Fig. 3d, we see that this transition probability is zero, indicating that the one-step mechanism is absent on anatase (101) surface as H atoms in H-O_t cannot proton-transferred from H_2O-Ti. In contrast, H can be transferred from the $H_2O^{(1)}$ to H-O_t (see the element marked by cyan triangle in Fig. 3d of the main text). The two-step mechanism

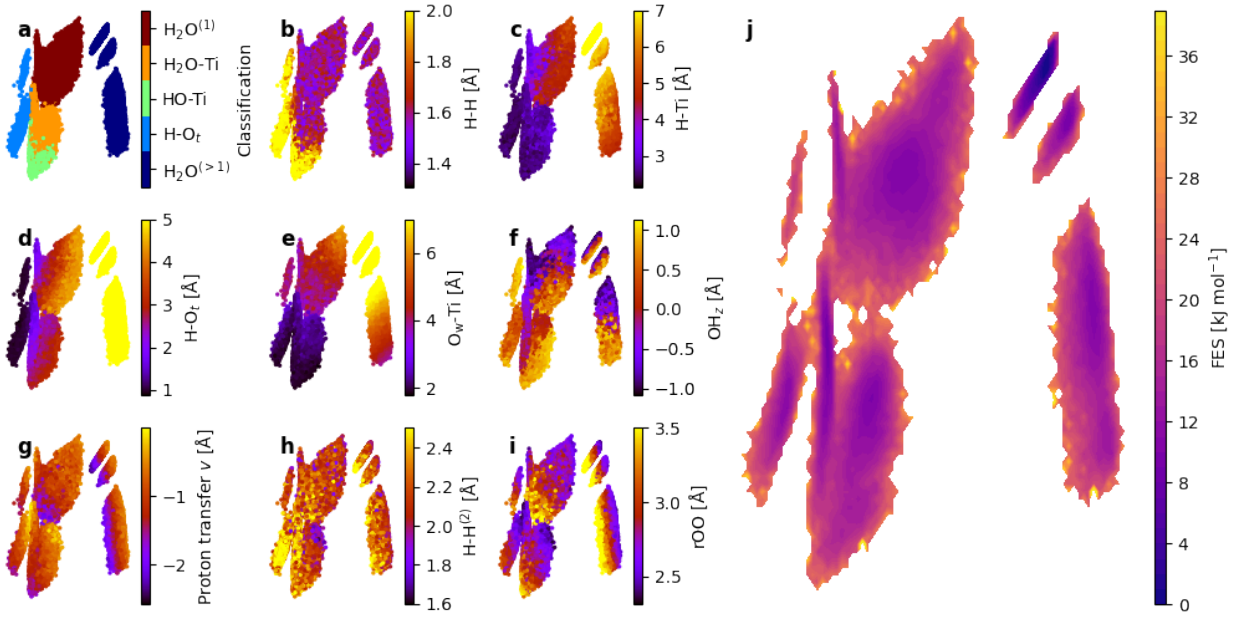

(e) rutile (011)

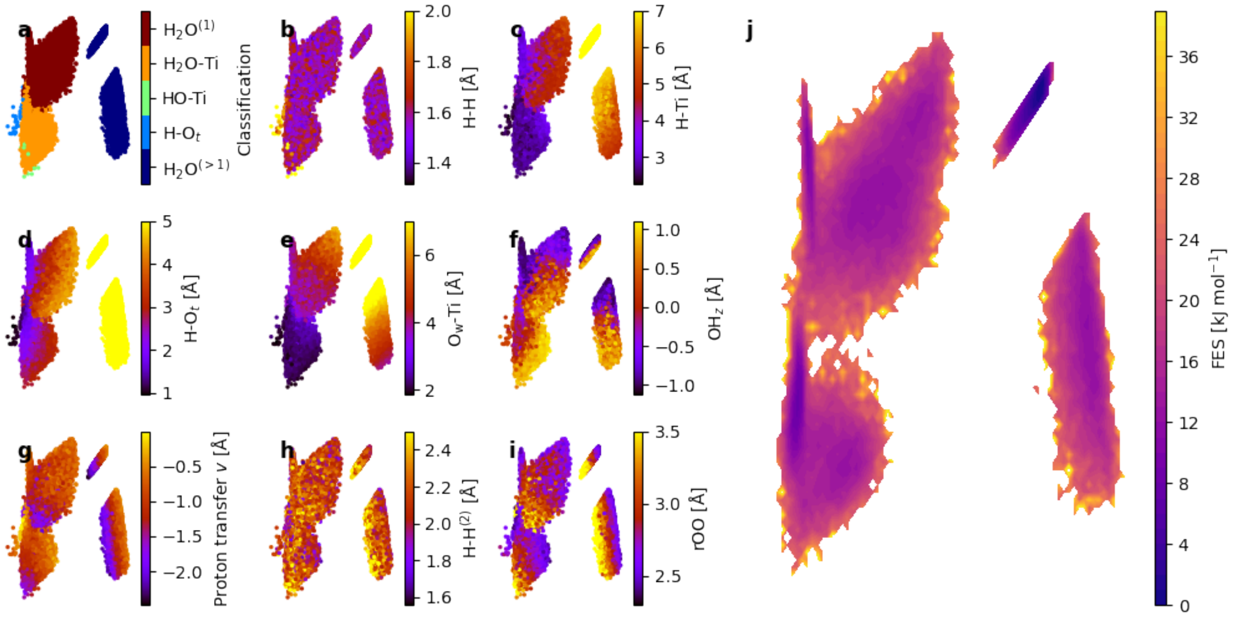

(f) rutile (100)

FIG. S11: (a-i) kernel Principal Component Analysis (kPCA) of the hydrogen atomic environments in various water-rutile TiO_2 systems. (j) The free energy surface (FES) as a function of the two principal axes of the kPCA map of the hydrogen environments (cont.).

($H_2O^{(1)}$ and H-O_t) thus dominates the proton transport and water dissociation on anatase (101) surface. This result also agrees well with the individual proton transfer pathways shown in Fig. 3b of the manuscript.

Fig. 3d of the main text shows the transition matrices between different states of hydrogen.

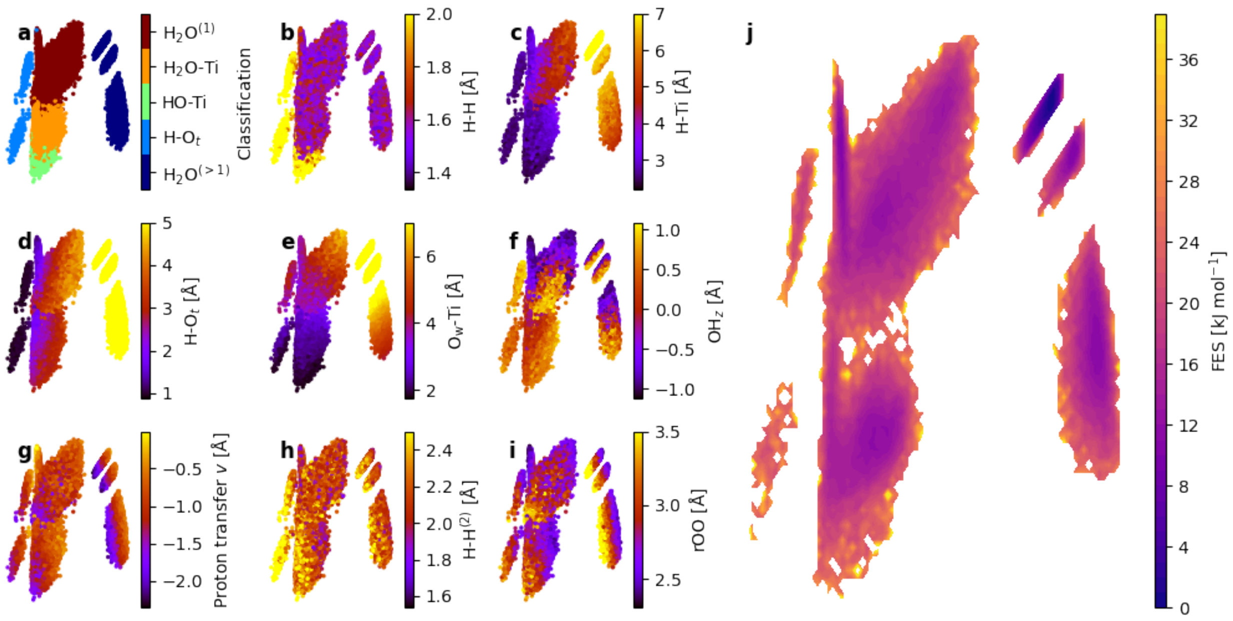

(g) rutile (110)

FIG. S11: (a-i) kernel Principal Component Analysis (kPCA) of the hydrogen atomic environments in various water-rutile TiO_2 systems. (j) The free energy surface (FES) as a function of the two principal axes of the kPCA map of the hydrogen environments (cont.).

The pathways for transitions are shown in Fig. 3c, where the two-step process involves the surface proton transport (dashed lines) and water dissociation pathways (solid lines). Here we introduce another method to distinguish water dissociation from proton transport, as well as to further validate the analysis in the main text.

This method uses proton tracking based on the nearest neighbour environment of oxygen atoms. Alg. 1 gives a brief pseudo-code overview of a basic algorithm for proton tracking using neighbour lists. In practice, it also requires some additions to filter out other kinds of surface proton transfers and determining the adsorption state of surface water. The code is provided in the SI repository.

Algorithm 1 Pseudo code for H tracing through proton transport

```
procedure HTracing(oxygen, counter=1)          ▹ Function to trace H through proton transport
▹ Get configurations in this and previous time step
currentNeighbours ← currentNL.getNeighbours(oxygen)            ▹ Current Configuration
previousNeighbours ← previousNL.getNeighbours(oxygen)          ▹ Previous Configuration

▹ Find atoms that have left from previousNeighbours
goneAtoms ← setDifference(currentNeighbours, previousNeighbours)
goneH ← findH(goneAtoms)                                       ▹ Filter out only H

newO ← currentNL.getNeighbours(goneH)          ▹ Find current configuration of departed H
newOConfig ← currentNL.getNeighbours(newO)           ▹ Find full configuration of that O

    if isOTi(newOConfig) then                  ▹ Terminate if H is in surface hydroxyl
return counter
    else                                       ▹ Otherwise do again until surface is found
        return HTracing(newO, counter=counter+1)
```

TABLE S4: Comparison of the hydrogen transition probability between different states on the anatase (100), (101) and (110) surfaces. The data is taken from Fig. 3d of the main text.

			anatase (100)		
	$H_2O^{(>1)}$	$H\text{-}O_t$	HO-Ti	H_2O-Ti	$H_2O^{(1)}$
$H_2O^{(>1)}$	9.81×10^{-1}	0	0	0	1.94×10^{-2}
$H\text{-}O_t$	0	1	0	0	9.83×10^{-4}
HO-Ti	0	0	9.28×10^{-1}	7.16×10^{-2}	0
H_2O-Ti	0	0	1.33×10^{-2}	9.80×10^{-1}	6.40×10^{-3}
$H_2O^{(1)}$	8.26×10^{-2}	6.33×10^{-5}	0	3.79×10^{-3}	9.14×10^{-1}
			anatase (101)		
	$H_2O^{(>1)}$	$H\text{-}O_t$	HO-Ti	H_2O-Ti	$H_2O^{(1)}$
$H_2O^{(>1)}$	9.64×10^{-1}	0	0	0	3.61×10^{-2}
$H\text{-}O_t$	0	1	0	0	2.26×10^{-4}
HO-Ti	0	0	1	1.71×10^{-4}	0
H_2O-Ti	0	0	1.69×10^{-4}	1	2.27×10^{-4}
$H_2O^{(1)}$	9.77×10^{-2}	1.60×10^{-4}	0	1.23×10^{-6}	9.02×10^{-1}
			anatase (110)		
	$H_2O^{(>1)}$	$H\text{-}O_t$	HO-Ti	H_2O-Ti	$H_2O^{(1)}$
$H_2O^{(>1)}$	9.80×10^{-1}	0	0	0	1.99×10^{-2}
$H\text{-}O_t$	0	1	1.87×10^{-5}	6.24×10^{-8}	0
HO-Ti	0	2.31×10^{-7}	7.27×10^{-1}	2.73×10^{-1}	0
H_2O-Ti	0	4.55×10^{-8}	1.00×10^{-1}	9.00×10^{-1}	0
$H_2O^{(1)}$	1.03×10^{-1}	0	0	0	8.97×10^{-1}

TABLE S5: Comparison of the hydrogen transition probability between different states on the rutile (001), (011), (100) and (110) surfaces. The data is taken from Fig. 3d of the main text.

rutile (001)					
	$H_2O^{(>1)}$	H-O_t	HO-Ti	H_2O-Ti	$H_2O^{(1)}$
$H_2O^{(>1)}$	9.54×10^{-1}	0	0	0	4.59×10^{-2}
H-O_t	0	9.87×10^{-1}	1.23×10^{-7}	1.29×10^{-2}	0
HO-Ti	0	3.72×10^{-6}	8.57×10^{-1}	1.43×10^{-1}	0
H_2O-Ti	0	3.03×10^{-2}	3.29×10^{-2}	9.37×10^{-1}	1.19×10^{-6}
$H_2O^{(1)}$	4.72×10^{-1}	0	0	2.54×10^{-7}	5.28×10^{-1}
rutile (011)					
	$H_2O^{(>1)}$	H-O_t	HO-Ti	H_2O-Ti	$H_2O^{(1)}$
$H_2O^{(>1)}$	9.74×10^{-1}	0	0	0	2.62×10^{-2}
H-O_t	0	9.02×10^{-1}	1.03×10^{-7}	9.77×10^{-2}	0
HO-Ti	0	7.82×10^{-7}	6.16×10^{-1}	3.84×10^{-1}	0
H_2O-Ti	0	2.49×10^{-2}	7.33×10^{-2}	8.82×10^{-1}	1.97×10^{-2}
$H_2O^{(1)}$	1.22×10^{-1}	0	0	8.55×10^{-10}	8.78×10^{-1}
rutile (100)					
	$H_2O^{(>1)}$	H-O_t	HO-Ti	H_2O-Ti	$H_2O^{(1)}$
$H_2O^{(>1)}$	9.82×10^{-1}	0	0	0	1.77×10^{-2}
H-O_t	0	5.33×10^{-2}	0	9.47×10^{-1}	0
HO-Ti	0	0	1.97×10^{-2}	9.80×10^{-1}	0
H_2O-Ti	0	2.25×10^{-4}	1.01×10^{-4}	1	5.24×10^{-6}
$H_2O^{(1)}$	7.09×10^{-2}	0	0	2.33×10^{-7}	9.29×10^{-1}
rutile (110)					
	$H_2O^{(>1)}$	H-O_t	HO-Ti	H_2O-Ti	$H_2O^{(1)}$
$H_2O^{(>1)}$	9.72×10^{-1}	0	0	0	2.82×10^{-2}
H-O_t	0	1	0	1.08×10^{-5}	3.83×10^{-4}
HO-Ti	0	0	9.53×10^{-1}	4.67×10^{-2}	0
H_2O-Ti	0	3.49×10^{-2}	3.47×10^{-2}	9.30×10^{-1}	3.91×10^{-4}
$H_2O^{(1)}$	2.42×10^{-2}	2.89×10^{-4}	2.73×10^{-8}	4.77×10^{-6}	9.75×10^{-1}

The results of the proton tracking algorithm are shown in Fig. S12. For all rutile surfaces, the one-step and two-step processes all originate from water dissociation. On anatase (101) two-step processes are dominated by water dissociation and have about 1 % surface proton transport. This is in agreement with previous results, which found these processes to be competing on anatase (101) [12]. Anatase (100) on the other hand shows exclusively two-step surface proton transport processes for the trajectory that was analyzed. In addition, it is worth noting that the algorithm can trace other surface proton transports events, for example, H atoms moving from one H-O_t site to another with no intermediate water molecules, or from one HO-Ti site to another with and without intermediate water molecules.

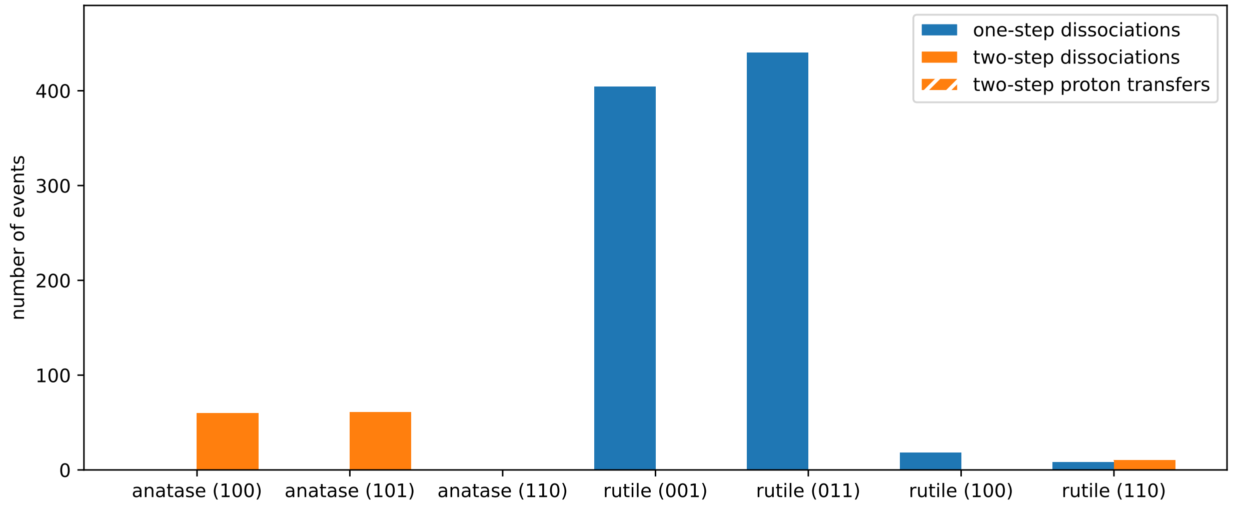

FIG. S12: Results of proton transfer algorithm for the seven TiO_2 surfaces. The number of proton transport events are shown as bar plots, where continuous bars are the number of dissociation events and surface proton transfer events are added as hatched bars on top. Blue bars denote one-step processes and orange bars two-step processes. If there is no bar for a certain surface type, no proton transports events are found.

SUPPLEMENTARY REFERENCES

[1] G. Lippert, J. Hutter, and M. Parrinello, "The Gaussian and augmented-plane-wave density functional method for ab initio molecular dynamics simulations," Theor. Chem. Acc. **103**, 124 (1999).

[2] T. T. Duignan, C. J. Mundy, G. K. Schenter, and X. S. Zhao, "Method for accurately predicting solvation structure," Journal of Chemical Theory and Computation **16**, 5401 (2020).

[3] Y. Yao and Y. Kanai, "Free energy profile of NaCl in water: First-principles molecular dynamics with SCAN and ωb97x-v exchange–correlation functionals," Journal of Chemical Theory and Computation **14**, 884 (2018).

[4] B. Wen, M. F. Calegari Andrade, L.-M. Liu, and A. Selloni, "Water dissociation at the water–rutile tio2 (110) interface from ab initio-based deep neural network simulations," Proceedings of the National Academy of Sciences **120**, e2212250120 (2023).

[5] N. H. Vu, H. V. Le, T. M. Cao, V. V. Pham, H. M. Le, and D. Nguyen-Manh, "Anatase–rutile phase transformation of titanium dioxide bulk material: a dft+ u approach," Journal of Physics: Condensed Matter **24**, 405501 (2012).

[6] M. Lazzeri, A. Vittadini, and A. Selloni, "Structure and energetics of stoichiometric tio 2 anatase surfaces," Physical Review B **63**, 155409 (2001).

[7] J. G. Lee, C. J. Pickard, and B. Cheng, "High-pressure phase behaviors of titanium dioxide revealed by a δ-learning potential," The Journal of chemical physics **156**, 074106 (2022).

[8] A. Reinhardt, C. J. Pickard, and B. Cheng, "Predicting the phase diagram of titanium dioxide with random search and pattern recognition," Physical Chemistry Chemical Physics **22**, 12697 (2020).

[9] F. Saidi, A. Mahmoudi, K. Laidi, T. Hidouri, and S. Nasr, "Structural, electronic and optical properties of m-doped anatase tio2 (m= fe or au): a first principle investigation," Computational Condensed Matter **28**, e00576 (2021).

[10] J. K. Mbae and Z. W. Muthui, "Ab initio investigation of the structural and electronic properties of alkaline earth metal-tio2 natural polymorphs," Advances in Materials Science and Engineering **2022** (2022).

[11] M. F. Calegari Andrade, H.-Y. Ko, R. Car, and A. Selloni, "Structure, polarization, and sum frequency generation spectrum of interfacial water on anatase tio2," The journal of physical chemistry letters **9**, 6716 (2018).

[12] M. F. C. Andrade, H.-Y. Ko, L. Zhang, R. Car, and A. Selloni, "Free energy of proton transfer at the water–tio 2 interface from ab initio deep potential molecular dynamics," Chemical Science **11**, 2335 (2020).

[13] V. Swamy, D. Menzies, B. C. Muddle, A. Kuznetsov, L. S. Dubrovinsky, Q. Dai, and V. Dmitriev, "Nonlinear size dependence of anatase ti o 2 lattice parameters," Applied Physics Letters **88**, 243103 (2006).

[14] L. Xu, C.-Q. Tang, J. Qian, and Z.-B. Huang, "Theoretical and experimental study on the electronic structure and optical absorption properties of p-doped tio2," Applied Surface Science **256**, 2668 (2010).

[15] R. Asahi, Y. Taga, W. Mannstadt, and A. J. Freeman, "Electronic and optical properties of anatase tio 2," Physical Review B **61**, 7459 (2000).

[16] D. Çakır and O. Gülseren, "First-principles study of thin tio x and bulklike rutile nanowires," Physical Review B **80**, 125424 (2009).

[17] B. Fu, G. Tang, and A. J. McGaughey, "Finite-temperature force constants are essential for accurately predicting the thermal conductivity of rutile tio 2," Physical Review Materials **6**, 015401 (2022).

[18] D. T. Cromer and K. Herrington, "The structures of anatase and rutile," Journal of the American Chemical Society **77**, 4708 (1955).

[19] K. Rościszewski, K. Doll, B. Paulus, P. Fulde, and H. Stoll, "Ground-state properties of rutile: Electron-correlation effects," Physical Review B **57**, 14667 (1998).

[20] B. Montanari and N. Harrison, "Lattice dynamics of tio2 rutile: influence of gradient corrections in density functional calculations," Chemical physics letters **364**, 528 (2002).

[21] F. Labat, P. Baranek, and C. Adamo, "Structural and electronic properties of selected rutile and anatase tio2 surfaces: an ab initio investigation," Journal of Chemical Theory and Computation **4**, 341 (2008).

[22] C. Schran, F. L. Thiemann, P. Rowe, E. A. Müller, O. Marsalek, and A. Michaelides, "Machine learning potentials for complex aqueous systems made simple," Proceedings of the National Academy of Sciences **118**, e2110077118 (2021).

[23] L. Zhang, J. Han, H. Wang, R. Car, and E. Weinan, "Deep potential molecular dynamics: a scalable model with the accuracy of quantum mechanics," Physical review letters **120**, 143001 (2018).

[24] L.-M. Liu, C. Zhang, G. Thornton, and A. Michaelides, "Structure and dynamics of liquid water on rutile tio 2 (110)," Physical Review B **82**, 161415 (2010).

[25] Y.-B. Zhuang, R.-H. Bi, and J. Cheng, "Resolving the odd–even oscillation of water dissociation at rutile tio2 (110)–water interface by machine learning accelerated molecular dynamics," The Journal of Chemical Physics **157**, 164701 (2022).
